# DNA damage in circulating leukocytes measured with the comet assay may predict the risk of death

**DOI:** 10.1038/s41598-021-95976-7

**Published:** 2021-08-18

**Authors:** Stefano Bonassi, Marcello Ceppi, Peter Møller, Amaya Azqueta, Mirta Milić, Monica Neri, Gunnar Brunborg, Roger Godschalk, Gudrun Koppen, Sabine A. S. Langie, João Paulo Teixeira, Marco Bruzzone, Juliana Da Silva, Danieli Benedetti, Delia Cavallo, Cinzia Lucia Ursini, Lisa Giovannelli, Silvia Moretti, Patrizia Riso, Cristian Del Bo’, Patrizia Russo, Malgorzata Dobrzyńska, Irina A. Goroshinskaya, Ekaterina I. Surikova, Marta Staruchova, Magdalena Barančokova, Katarina Volkovova, Alena Kažimirova, Bozena Smolkova, Blanca Laffon, Vanessa Valdiglesias, Susana Pastor, Ricard Marcos, Alba Hernández, Goran Gajski, Biljana Spremo-Potparević, Lada Živković, Elisa Boutet-Robinet, Hervé Perdry, Pierre Lebailly, Carlos L. Perez, Nursen Basaran, Zsuzsanna Nemeth, Anna Safar, Maria Dusinska, Andrew Collins, for the hCOMET project, Diana Anderson, Vanessa Andrade, Cristiana Costa Pereira, Solange Costa, Kristine B. Gutzkow, Carina Ladeira, Massimo Moretti, Carla Costa, Irene Orlow, Emilio Rojas, Bertrand Pourrut, Marcin Kruszewski, Siegfried Knasmueller, Sergey Shaposhnikov, Bojana Žegura, Helga Stopper

**Affiliations:** 1 Unit of Clinical and Molecular Epidemiology, IRCCS San Raffaele Roma, Rome, Italy; 2 Department of Human Sciences and Quality of Life Promotion, San Raffaele University, Unit of Clinical and Molecular Epidemiology, IRCCS San Raffaele Roma, Via di Val Cannuta, 247, 00166 Rome, Italy; 3 Clinical Epidemiology Unit, San Martino Policlinic Hospital, Genoa, Italy; 4 Department of Public Health, Section of Environmental Health, University of Copenhagen, Oster Farimagsgade 5A, 1014 Copenhagen, Denmark; 5 Department of Pharmacology and Toxicology, University of Navarra, C/Irunlarrea 1, 31008 Pamplona, Spain; 6 C/Irunlarrea 3, IdiSNA, Navarra Institute for Health Research, 31008 Pamplona, Spain; 7 Mutagenesis Unit, Institute for Medical Research and Occupational Health, Ksaverska cesta 2, 10000 Zagreb, Croatia; 8 Department of Environmental Health, Section of Molecular Toxicology, Norwegian Institute of Public Health (NIPH), Lovisenberggt 6, 0456 Oslo, Norway; 9 Department of Pharmacology and Toxicology, School of Nutrition and Translational Research in Metabolism, University of Maastricht, Universiteitssingel 50, 6200 MD Maastricht, The Netherlands; 10 Flemish Institute of Technological Research, Environmental Risk and Health Unit VITO - BIOMo, Mol, Belgium; 11 Environmental Health Department, National Institute of Health, Rua Alexandre Herculano, 321, 4000-055 Porto, Portugal; 12 Environmental Health Department, Instituto Nacional de Saúde Doutor Ricardo Jorge, Rua Alexandre Herculano 321, 4000-055 Porto, Portugal; 13 EPIUnit - Instituto de Saúde Pública, Universidade Do Porto, Rua das Taipas, no 135, 4050-600 Porto, Portugal; 14 Laboratory of Genetic Toxicology, Lutheran University of Brazil (ULBRA), and La Salle University (UNILASALLE), Canoas, RS Brazil; 15 Department of Occupational and Environmental Medicine, Epidemiology and Hygiene (DiMEILA), Italian Workers’ Compensation Authority (INAIL), Via Fontana Candida 1, 00078 Monte Porzio Catone (Rome), Italy; 16 Department NEUROFARBA, University of Florence, Viale G. Pieraccini 6, 50139 Florence, Italy; 17 Department of Health Sciences, Division of Dermatology, University of Florence, Palagi Hospital, Viale Michelangelo 41, Florence, Italy; 18 Department of Food, Environmental and Nutritional Sciences (DeFENS), University of Milan, Via Celoria 2, 20133 Milan, Italy; 19 Department of Radiation Hygiene and Radiobiology, National Institute of Public Health NIH – National Research Institute, 24 Chocimska Street, 00-791 Warsaw, Poland; 20 Laboratory for the Study of the Pathogenesis of Malignant Tumors, National Medical Research Center for Oncology, 14 line 63, 344037 Rostov-on-Don, Russia; 21 Institute of Biology, Medical Faculty, Slovak Medical University, Limbova 12, 83303 Bratislava, Slovakia; 22 Cancer Research Institute, Biomedical Research Center of the Slovak Academy of Sciences, Dubravska cesta 9, Bratislava, Slovakia; 23 Grupo DICOMOSA, Centro de Investigaciones Científicas Avanzadas (CICA), Departamento de Psicología, Facultad de Ciencias de La Educación, Universidade da Coruña, Campus Elviña s/n, 15071 A Coruña, Spain; 24 Instituto de Investigación Biomédica de A Coruña (INIBIC), AE CICA-INIBIC, Oza, 15071 A Coruña, Spain; 25 Grupo DICOMOSA, Centro de Investigaciones Científicas Avanzadas (CICA), Departamento de Biología, Facultad de Ciencias, Universidade da Coruña, Campus A Zapateira s/n, 15071 A Coruña, Spain; 26 Department of Genetics and Microbiology, Faculty of Biosciences, Universitat Autònoma de Barcelona, 08193 Cerdanyola del Vallès (Barcelona), Spain; 27 Consortium for Biomedical Research in Epidemiology and Public Health (CIBERESP), Carlos III Institute of Health, 28029 Madrid, Spain; 28 Center of Biological Research, Faculty of Pharmacy, University of Belgrade, VojvodeStepe 450, Belgrade, Serbia; 29 Toxalim (Research Centre in Food Toxicology), Université de Toulouse, INRAE, ENVT, INP-Purpan, UPS, Toulouse, France; 30 Univ Paris-Saclay, CESP, Villejuif, France; 31 ANTICIPE Unit, INSERM & University of Caen-Normandie Centre François Baclesse, Avenue du Général Harris, 14076 Caen Cedex 05, France; 32 Department of Biochemistry, Instituto de Ciencias Básicas Y Preclínicas “Victoria de Giron”, Universidad de Ciencias Médicas de La Habana, 146 St. and 31 Ave, No, 3102 Playa, Habana, Cuba; 33 Department of Pharmaceutical Toxicology, Faculty of Pharmacy, Hacettepe University, Ankara, Turkey; 34 Department of Non-Ionizing Radiation, National Public Health Center, Anna Street 5, 1221 Budapest, Hungary; 35 NILU, Health Effects Laboratory, Kjeller, Norway; 36 Department of Nutrition, Institute of Basic Medical Sciences, University of Oslo, Sognsvannsveien 9, 0372 Oslo, Norway; 37 University of Bradford, Bradford, UK; 38 University of Southern Santa Catarina, Santa Catarina, Brazil; 39 National Institute of Health Doutor Ricardo Jorge, Porto, Portugal; 40 Norwegian Institute of Public Health, Olso, Norway; 41 Instituto Politécnico de Lisboa, Universidade NOVA, Lisbon, Portugal; 42 University of Perugia, Perugia, Italy; 43 Memorial Sloan Kettering Cancer Center, New York, USA; 44 Universidad Nacional Autónoma de México, Mexico City, Mexico; 45 ISA Lille, Université de Toulouse, Toulouse, France; 46 Institute of Nuclear Chemistry and Technology, Warszawa, Poland; 47 Medical University of Vienna, Wien, Austria; 48 University of Oslo, Olso, Norway; 49 National Institute of Biology, Ljubljana, Slovenia; 50 University of Würzburg, Würzburg, Germany

**Keywords:** Biomarkers, Risk factors

## Abstract

The comet assay or single cell gel electrophoresis, is the most common method used to measure strand breaks and a variety of other DNA lesions in human populations. To estimate the risk of overall mortality, mortality by cause, and cancer incidence associated to DNA damage, a cohort of 2,403 healthy individuals (25,978 person-years) screened in 16 laboratories using the comet assay between 1996 and 2016 was followed-up. Kaplan–Meier analysis indicated a worse overall survival in the medium and high tertile of DNA damage (p < 0.001). The effect of DNA damage on survival was modelled according to Cox proportional hazard regression model. The adjusted hazard ratio (HR) was 1.42 (1.06–1.90) for overall mortality, and 1.94 (1.04–3.59) for diseases of the circulatory system in subjects with the highest tertile of DNA damage. The findings of this study provide epidemiological evidence encouraging the implementation of the comet assay in preventive strategies for non-communicable diseases.

## Introduction

The induction of DNA damage following exposure to exogenous and endogenous agents is an important first step in the process of carcinogenesis. Replication of damaged DNA may lead to mutations or structural changes to the chromosomes, events which are critical in the development of cancer^1^. The induction of genome instability resulting from these events is considered to be one of the critical cancer hallmarks^2, 3^. In addition, most non-communicable diseases (NCDs)—leading causes of death and disability—show characteristic phenotypes, e.g., accelerated aging, which are the result of accumulated DNA damage, telomere capping loss, and oxidative stress. DNA damage caused by oxidative reactions plays a key role in the development of NCDs^4^. The most common method for measuring DNA damage in human populations is the comet assay, or single cell gel electrophoresis. In brief, agarose-embedded cells are lysed, leaving histone-depleted nuclei known as nucleoids, containing supercoiled DNA; breaks in the DNA relax supercoiling and the relaxed DNA loops are free to migrate under electrophoresis, forming a structure resembling a comet, the relative DNA content of the comet tail indicating the frequency of breaks^4–6^. Because of its simplicity, sensitivity and speed, it has been widely adopted as a biomarker assay in human studies. Monitoring occupational exposure to mutagens, testing dietary supplementation with antioxidants, or checking levels of oxidative stress in relation to diverse diseases are among the most common objectives in human studies with the comet assay^5–7^. Extensive literature has been published on the assay (see references ^5–7^ for a description of the protocol). The assay is generally used as a marker of exposure, but given the wide range of events associated with DNA damage, the comet assay is well suited to evaluate the interaction between lifestyle factors, environmental exposures and genetic background, and as a consequence to contribute to risk assessment and the surveillance of populations at risk of NCDs. Indeed, cross-sectional studies have demonstrated that subjects with the most prevalent NCDs have elevated levels of DNA damage, measured by the comet assay, in circulating leukocytes^5, 7^. However, an association of DNA damage with disease does not mean that DNA damage is the cause of disease; it could instead be an effect. The higher level of chromosome damage in cancer patients, or the pre-mortem lowering of systolic blood pressure are paradigmatic examples of this bias^8, 9^. Whether the level of DNA damage in healthy individuals is a potential marker of individual risk of cancer and other NCDs is a question best resolved through cohort studies, which circumvent this problem of reverse causality. The historical cohort design, i.e., those studies in which data on the relevant events for each individual (in this case the results of assays measuring genetic damage) are collected from existing records and can immediately be analyzed, has proven to be the best approach for the validation of genetic biomarkers as risk predictors. Examples of biomarkers of genomic instability that have been linked to the risk of cancer with this study design include the frequency of chromosomal aberrations and micronuclei in peripheral lymphocytes^10–16^. Other biomarkers such as DNA adducts have been positively investigated using a nested case–control design with analysis of stored samples^17, 18^, although the historical cohort approach remains the most appropriate for those biomarkers that are preferably evaluated on fresh tissue.

Modern epidemiological study of cancer and other chronic diseases depends increasingly on the use of large datasets assembled within the framework of international collaborative studies. This is also the case of the ComNet initiative which started in 2011 gathering a network of over 100 laboratories working with the comet assay^5, 18^. ComNet is now subsumed in the COST Action hCOMET, which has created a database consisting of individual comet assay data from nearly 20,000 subjects, contributed from 105 studies performed in 44 laboratories between 1999 and 2019. A detailed description of the database, including the list of participating laboratories and the baseline frequency of the assay descriptors has been recently published^19^. All laboratories contributing data to the hCOMET database were invited to participate to a historical cohort study, assuming that they had access to individual data required for a follow-up of cancer incidence and mortality, and that—after receiving ethical approval—they could interrogate local/national cancer registries or registries of causes of death.

The aim of the hCOMET cohort study was to provide an estimate of the association between DNA damage—as measured by the standard comet assay in healthy individuals—and overall mortality, mortality by cause, and cancer incidence for those laboratories with access to cancer registry data.

### Results

To estimate the risk of overall mortality, mortality by cause, and cancer incidence associated with DNA damage, a cohort of 2,403 healthy individuals (25,978 person-years) screened in 16 laboratories using the comet assay between 1996 and 2016 was followed-up. Overall, 308 deaths were registered by the end of the follow-up period (median duration 10.8 years). The most common causes of death were malignant neoplasms (108), and diseases of the circulatory system (77). As regards cancer, gastro-intestinal (20), and genito-urinary (31) were the most frequent locations. One hundred and fifty-one cancer cases were recorded, mostly from laboratories EU31 (31 cases) and EU33 (101 cases).

**Figure 1:**
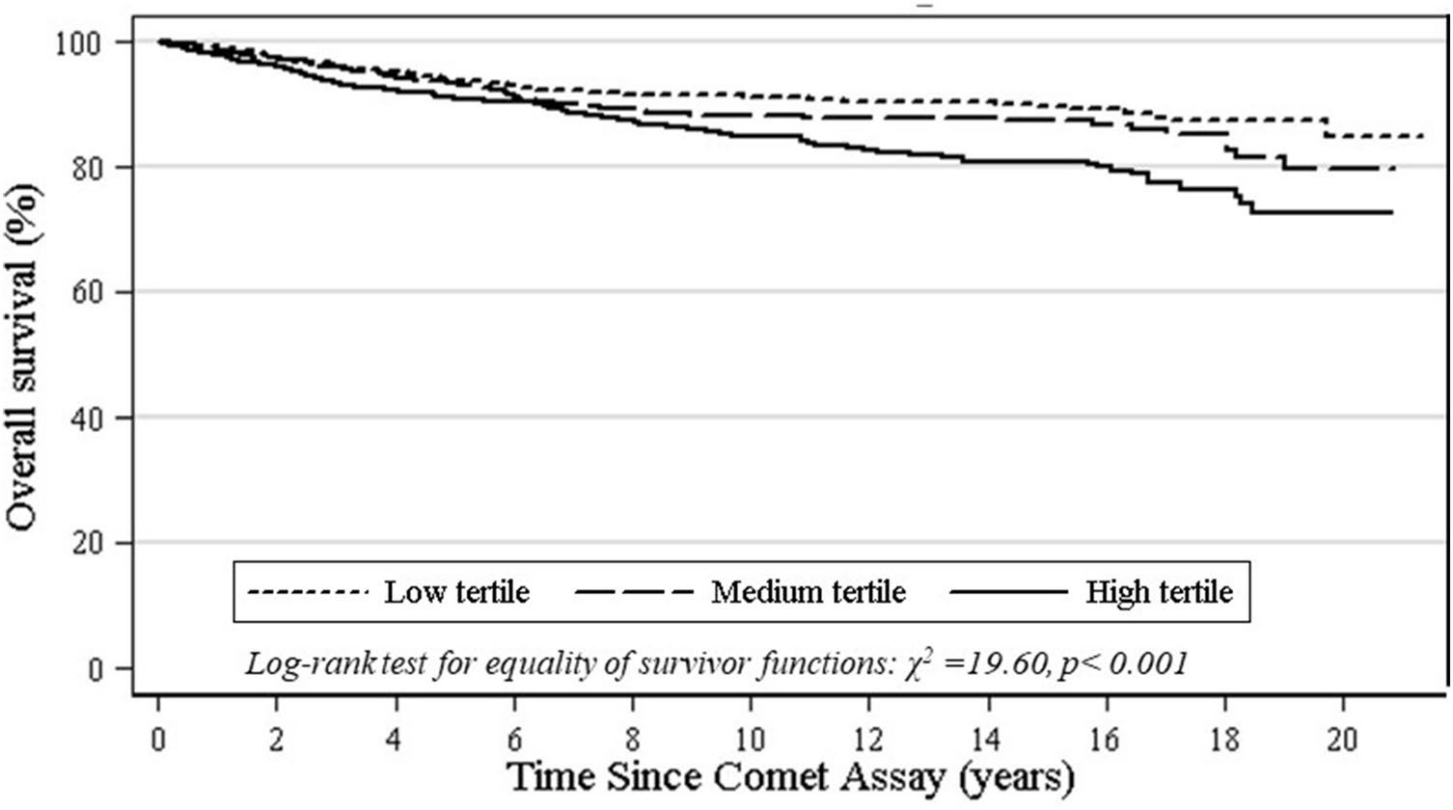
Kaplan–Meier curve for overall mortality by tertile of DNA damage (composite endpoint) measured with the comet assay. The hCOMET cohort.

**Figure 2:**
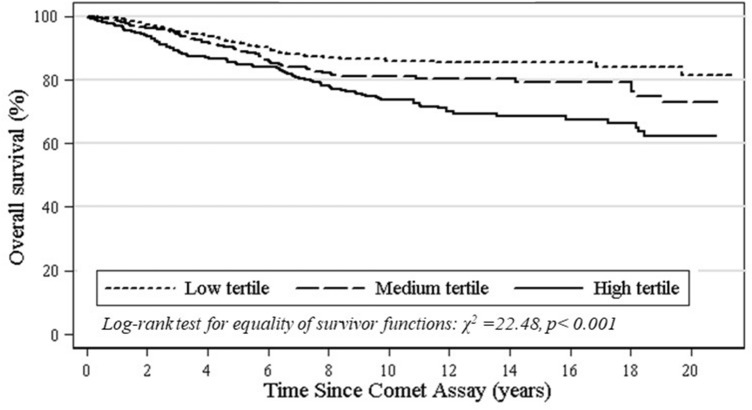
Kaplan–Meier curve for overall mortality by tertile of DNA damage (%T) measured by the Comet assay. The hCOMET cohort.

The DNA damage level measured by the comet assay is reported as DNA migration, using either length of the comet tail (TL), percentage of fluorescence of DNA in the comet tail (%T), the product of the TL and %T (i.e. tail moment, TM) or visual score (VS, based on categorization of the comets into different classes) as primary comet assay descriptors. A preliminary comparison of DNA damage levels between the groups of deceased, incident cancer cases, and subjects still alive is reported in Tables 1, 2. Mean values of the comet descriptors TM, TL, VS and %T were higher for the groups of deceased and cancer cases, although a statistically significant difference with subjects alive was only reached for %T in deceased (18.0 *vs* 9.0; *P* < 0.001) and in cancer cases (17.7 *vs* 12.4; *P* < 0.019), respectively. The association between mortality risk and tertile of DNA damage was further investigated with the Kaplan–Meier analysis, which indicated a worse outcome in subjects in the medium or high tertile of DNA damage, a result which was consistent over the follow-up time, as shown by Fig. 1 (composite endpoint; χ^2^_2_ = 19.6, *P* < 0.001) and Fig. 2 (%T; χ^2^_2_ = 22.48, *P* < 0.001).

**Table 1:** Selected characteristics of national cohorts. The hCOMET cohort study.

Country	Lab code	Subjects	Deaths	Cancer cases	Person years	Median follow-up (years)	Period of test	Mean age at test (SD)	Males (%)	Exposed (%)	Smokers (%)
Cuba	CSA2	71	0	na	242	3.4	2015	44.4 (10.5)	87.3	0.0	57.8
Brazil	CSA7	199	7	5	1,584	9.5	2008–2014	45.4 (13.7)	100.0	66.3	0.0
Italy	EU15	105	2	2	1,450	15.2	2002–2007	36.4 (8.3)	29.5	28.6	44.8
Italy	EU17	64	5	na	802	13.2	2002–2006	46.1 (15.9)	39.1	67.2	4.7
Italy	EU18	59	0	1	578	10.1	2006–2013	29.3 (12.1)	100.0	0.0	91.5
Italy	EU19	77	36	na	219	3.0	2013–2015	72.1 (8.8)	45.5	100.0*	77.9
Poland	EU23	86	1	na	498	5.8	2012	44.0 (11.1)	19.8	53.5	44.2
Russia	EU27	17	0	na	45	3.0	2015–2016	52.6 (13.5)	0.0	Na	23.5
Slovakia/ Norway	EU31	738	22	31	10,403	14.3	1996–2009	43.0 (15.6)	51.4	60.0	25.5
Spain	EU32	124	2	5	1,280	8.9	2003–2010	42.5 (12.3)	57.3	63.7	40.3
Spain	EU33	507	217	101	3,649	7.7	2007–2013	63.1 (15.1)	62.1	100.0*	44.0
Turkey	EU34	30	0	na	600	20.0	1998	28.7 (5.4)	0.0	100.0	46.7
Croatia	EU4	105	0	na	1,248	12.8	2006–2008	39.6 (10.2)	59.1	46.7	38.1
Serbia	EU42	32	1	na	125	4.2	2014	63.4 (11.7)	21.9	100.0*	37.5
Hungary	EU50	37	0	na	433	12.0	2002–2012	38.4 (11.1)	64.9	0.0	na
France	EU8	152	15	6	2,822	18.9	1997–2000	42.4 (11.2)	100.0	100.0	0.0
Total		2,403	308	151	25,978	10.8	1996–2016	47.8 (17.1)	59.8	67.4	32.2

na: not available; *NCD Patients.

**Table 2:** Comparison of subjects diagnosed with cancer, deceased for any cause, and alive according to mean DNA damage level measured by the main descriptors of the comet assay. The hCOMET cohort.

Vital status	Tail moment		Tail length		Tail intensity %		Visual scoring	
	No	Mean ± SD	No	Mean ± SD	No	Mean ± SD	No	Mean ± SD
Alive	233	9.1 (14.9)	316	21.8 (13.7)	956	9.0 (10.2)	1,081	73.2 (61.6)
Deceased	3	15.7 (19.3)	3	22.7 (14.1)	244	18.0 (12.8)*	66	187.2 (109.3)
Cancer- free	103	20.1 (17.1)	208	25.4 (15.4)	790	12.4 (12.0)	842	61.1 (42.5)
Cancer-cases	2	15.9 (9.1)	6	28.4 (17.2)	115	17.7 (12.5)**	36	68.7 (44.0)

To ensure comparability, t-test was performed on standardized measures at laboratory and study level; * P < 0.001 (vs alive);** P = 0.017 (vs cancer free*).*

A Cox’s proportional hazard model evaluating the association between overall mortality and tertile of DNA damage was applied to the whole database, and results are reported in Table 3. The adjusted Hazard Ratio (HR) increased with the tertile of DNA damage (p for trend = 0.02) reaching statistical significance for subjects in the highest tertile when compared with the lowest tertile (HR = 1.42; 95% CI = 1.06–1.90; *P* = 0.02). Most of the other parameters included in the model, including age, smoking habit, and the presence of NCDs, showed significantly increased mortality risks, providing an intrinsic validation to study findings. Not surprisingly, the presence of NCD was the strongest predictor of mortality (HR = 13.2; 95% CI = 7.12–24.5; *P* < 0.001). The test for interaction and the consistency in all strata of the risk for the highest tertile of DNA damage (Table 4), confirmed that the presence of genotoxic exposure or a chronic disease did not modify the association between DNA damage and mortality rates. A complementary analysis of data shown in Table 4 using the medium tertile in the group of unexposed controls as the referent value for the interaction analysis confirmed (although on the basis of small numbers) that also in this group subjects in the highest tertile of DNA damage had the highest risk of death (HR = 6.07; 1.33–27.7; *P* = 0.02) (Supplementary Table 1). Increasing trends of HR were found also in occupationally exposed subjects, and especially in subjects affected by chronic diseases. An additional analysis evaluated the association between DNA damage and mortality risk after removing deaths due to accidents. The association was a little stronger, with a HR = 1.25; 0.90–1.73 for the medium tertile and a HR = 1.52: 1.11—2.08 (p = 0.010), for the highest tertile. No effect modification due to smoking habit could be found (p = 0.959), and no increase due to interaction was found in the group of subjects in the highest tertile of DNA damage who smoked. To evaluate the consistency of the observed effects over the different laboratories, a cohort-specific analysis was performed. The small number of events in most cohorts allowed a proper analysis only in a few datasets. Results referring to groups with more than 10 deaths, i.e., EU19, EU31, EU33, and EU8 are reported in Table 5. An increased mortality risk associated with DNA damage was present for the highest tertile in all cohorts but EU19, although no single laboratory reached statistical significance. To evaluate if the overall association between DNA damage and mortality was driven by a specific cohort, a sensitivity analysis was performed, sequentially removing a cohort from the analysis. The HR for the highest tertile of DNA damage, ranging from 1.33 to 1.51, remained significantly increased except when EU17, EU31 and EU33 were removed, though no major changes in the HR were observed.

**Table 3:** Overall mortality risk by tertile of DNA damage measured with the comet assay (composite endpoint), age, sex, smoking habit, and occupational exposure/disease. The hCOMET cohort.

	Deaths (no.)	Subjects (no.)	Hazard ratio	95% confidence interval	*P* value
DNA damage					
*Low tertile*	78	829	1	-	
*Medium tertile*	96	792	1.24	0.91–1.68	0.176
*High tertile*	134	782	1.42	1.06–1.90	0.02
Age at test *(years)*	308	2,403	1.09	1.07–1.10	< 0.001
Sex					
*Females*	107	965	1	-	
*Males*	201	1,438	1.17	0.89–1.53	0.253
Smoking					
*Non-smokers*	139	1,478	1	-	
*Smokers*	148	774	1.43	1.09–1.88	0.01
Exposure					
*Controls*	13	781	1	-	
*Occupationally exposed*	41	989	1.28	0.64–2.56	0.488
*NCD patients*	254	616	13.2	7.12–24.5	< 0.001

For some predictors the sum of the frequencies of each category does not amount to the overall due to missing values.

**Table 4:** Overall mortality risk by tertile of DNA damage measured with the comet assay (composite endpoint). Interaction analysis by occupational exposure/disease. The hCOMET cohort.

	Deaths (no.)	Subjects (no.)	Hazard ratio	95% confidence interval	*P* value
Controls	13	781	1.00	-	-
Occupationally exposed					
*Low tertile*	12	349	1.10	0.47–2.55	0.828
*Medium tertile*	12	314	1.18	0.51–2.73	0.704
*High tertile*	17	326	1.52	0.70–3.34	0.291
NCD patients					
*Low tertile*	65	208	11.2	5.75–21.7	< 0.001
*Medium tertile*	82	204	14.1	7.42–26.8	< 0.001
*High tertile*	107	204	14.5	7.72–27.3	< 0.001

Estimates adjusted for age, sex, smoking habit. For some predictors the sum of the frequencies of each categories does not amount to the overall due to missing values.

**Table 5:** Mortality risk by laboratory and tertile of DNA damage measured with the comet assay (composite endpoint). Laboratories with at least 10 deaths were considered. The hCOMET cohort.

	Deaths (no.)	Subjects (no.)	Hazard ratio	95% confidence interval	P value
EU19					
*Low tertile*	14	27	1	-	
*Medium tertile*	11	25	0.72	0.31–1.68	0.447
*High tertile*	11	25	0.97	0.41–2.32	0.946
EU31					
*Low tertile*	6	250	1	-	
*Medium tertile*	5	245	0.89	0.27–2.99	0.855
*High tertile*	11	243	1.82	0.67–4.97	0.243
EU33					
*Low tertile*	51	169	1	-	
*Medium tertile*	71	169	1.28	0.88–1.85	0.19
*High tertile*	95	169	1.3	0.91–1.86	0.147
EU8					
*Low tertile*	3	60	1	-	
*Medium tertile*	5	46	1.01	0.22–4.58	0.997
*High tertile*	7	46	1.58	0.39–6.35	0.522

Estimates adjusted for age, sex, smoking habit, and occupational exposure.

The HR for the medium tertile ranged from 0.77 to 1.31, was never significant, and was only once below 1.0, when EU33 was removed. The analysis for specific causes of death showed the strongest association for the sub-group of gastro-intestinal cancers (medium tertile; HR = 3.63; 0.71–18.50, and high tertile; HR = 7.45: 1.55–35.8, *P* = 0.012). In the group of genito-urinary cancers, the association was more evident for prostate and bladder cancers (high tertile; HR = 1.99: 0.76–5.20). A significant association with diseases of the circulatory system was found with the highest tertile of DNA damage (high tertile; HR = 1.94; 1.04–3.59, *P* = 0.036) (Table 6). In general, the small number of events limited the statistical power of these comparisons and the causes of death that could be studied.

**Table 6:** Mortality risk by selected causes of death and tertile of DNA damage measured with the comet assay (composite endpoint). The hCOMET cohort.

	Deaths (no.)	Hazard ratio	95% confidence interval	*P* value
All neoplasms (140–239)				
*Low tertile*	25	1	-	-
*Medium tertile*	37	1.46	0.87–2.45	0.153
*High tertile*	46	1.53	0.92–2.55	0.104
Gastro-intestinal neoplasms (150–157)				
*Low tertile*	2	1	-	-
*Medium tertile*	6	3.63	0.71–18.5	0.121
*High tertile*	12	7.45	1.55–35.8	0.012
Prostate and bladder neoplasms (185,188)				
*Low tertile*	6	1	-	-
*Medium tertile*	10	1.68	0.60–4.69	0.32
*High tertile*	15	1.99	0.76–5.20	0.159
Diseases of the circulatory system (390–459)				
*Low tertile*	18	1	-	-
*Medium tertile*	23	1.48	0.78–2.80	0.23
*High tertile*	36	1.94	1.04–3.59	0.036

Estimates adjusted for age, sex, smoking habit, and occupational exposure. In brackets ICD-9 (International Classification of Diseases-9) codes.

To investigate if the proximity to the end of follow-up might have affected the strength of the association a sensitivity analysis was performed stratifying the results by distance from the final event using a cut-off of five years. The HR for the highest tertile of DNA damage was consistent in both periods, i.e., HR = 1.36; 0.93–2.00, and HR = 1.53; 0.96–2.44, respectively (see Supplementary Table 2).

### Discussion

The results of the present study provide support for the hypothesis that an increased level of DNA damage represents a relevant event in the pathway leading to chronic disease and eventually to death. In addition, the use of circulating leukocytes suggests that DNA damage can be suitably measured in this surrogate tissue to estimate mortality risk. The observation that the association between DNA damage and risk of death is not dependent on the proximity with the outcome, i.e., is not driven by the possible presence of the disease, is consistent with the hypothesis that measuring DNA damage in healthy subjects at any time may predict NCD and death. The small size of the cohort calls for longer follow-up period, and the heterogeneity of the primary comet assay descriptors suggests that further insight into collinearity may reduce the risk of misclassification.

Genome instability represents an enhanced accumulation of mutations, and has been recognized as a hallmark of several chronic diseases and ageing^20^. The comet assay is considered among the most suitable methods for monitoring DNA damage in population studies. It is a simple, fast, accurate, inexpensive method that requires small sample volumes and can be performed on frozen blood or buffy coat^25^. This assay fulfils all criteria for routine use in large-scale molecular epidemiology studies and clinical disease management, especially with the recent development of high throughput versions; in addition, modifications of the protocol allow the detection of a variety of lesions and DNA repair activity^21^. There have been many reports over the years of elevated DNA damage levels (strand breaks and/or oxidized bases) associated with a wide range of clinical conditions such as coronary artery disease, diabetes, kidney disease, chronic obstructive pulmonary disease, multiple sclerosis, and Alzheimer's disease^4, 7, 22–25^. DNA damage and DNA repair are important elements in the etiology of cancer and in its treatment; Vodicka and colleagues^26^ reviewed the DNA damage information available for 17 types of cancer emphasizing the sensitivity and cost-effectiveness of the comet assay in evaluating DNA damage. Several studies have been reported on inflammatory disorders, e.g., arthritis, inflammatory bowel disease, where DNA oxidation damage is often accompanied by depressed antioxidant status^27, 28^. Russo et al. have tested the potential application of the comet assay as a prognostic tool for patients with chronic obstructive pulmonary disease (COPD) undergoing pulmonary rehabilitation^25^. Likewise, Corredor et al. demonstrated in a cohort of hemodialysis patients that deceased subjects at the end of the follow-up period (approximately four years) had higher level of DNA strand breaks at baseline, i.e., at the entry of the study, than subjects still alive^29^. Despite the strong evidence from cross-sectional studies linking the presence of several chronic diseases with an increased level of DNA damage, and the extensive literature on mechanisms, clearly showing that elevated levels of DNA damage are associated with several NCDs, it remains a challenge to prove that DNA damage is a cause rather than an effect of the disease. The most suitable epidemiologic approach to evaluate long-term effects when the biomarker may be affected by the disease, i.e., reverse causality bias, is the cohort study, generally with a non-concurrent design when biomarkers are involved.

The observation reported here of an association between DNA damage and overall mortality lends support to a possible use of DNA damage as risk biomarker, envisioning public health implications, environmental and occupational preventive strategies, and even diagnostic use in clinics. The higher risks of death observed for those covariates known to be associated with mortality, i.e., age, smoking habit, and the presence of chronic diseases, gave an intrinsic validation to results on DNA damage biomarkers. The use of a composite endpoint represented by the tertile of any descriptors measured in the original studies (when tail intensity was not available) is fully justified by the common target of different descriptors, i.e., measuring the extent of DNA damage. This choice was further supported by 1) the presence of correlation between different descriptors of the comet assay reported in the literature and confirmed in the hCOMET dataset, and 2) the substantial overlapping of results between %T and the composite endpoint. On the other hand, the positive association between the composite endpoint and the risk of death and NCD may have been attenuated by the use of TL, which in many instances has been demonstrated to have a lower sensitivity in identifying DNA damage by known genotoxic agents.^19^. The underlying explanation is that tail length increases with DNA break frequency only up to a certain point – corresponding to a quite low level of damage. This reflects the fact that the comet tail is formed by broken DNA loops extending towards the anode, and so the tail length – once established – is defined by the length of the DNA loops. In most laboratories using software image analysis, the TL has a rather narrow dose–response relationship for direct strand-breaking agents such as ionizing radiation^30^. The dynamic range of the TM is mainly influenced by %T as the TL does not differ much. Thus, the TM typically shows a linear dose–response relationship with ionizing radiation^31, 32^^.^

A major concern was the possibility that the overall association with mortality was modified by the presence of exposure to DNA-damaging agents, or by the presence of disease. Although this hypothesis can be properly checked only with ad hoc studies^11, 33^, the interaction analysis reported in Table 4 and Supplementary Table 1 suggests that an increasing trend in the risk of death by tertile of DNA damage was present in all groups of subjects evaluated, i.e., non-exposed subjects, occupationally exposed subjects, and NCD patients. As regards smoking, the lack of interaction with DNA damage level makes it unlikely that the higher risk of death associated to the tertile of DNA damage could be explained by this habit. Another potential source of confounding or of effect modification was the presence of several sub-cohorts. This issue was addressed with a sensitivity analysis, which demonstrated that no single sub-cohort could change the overall results.

The presence of selection bias is a critical issue in retrospective cohort studies. Subjects may know about their condition, and in some cases, subjects exposed to the disease have a higher interest in participation, especially if the study question was controversial. This may result in a biased assessment or in a selective loss to follow-up associated to exposure. This is hardly the case in the present study, not only because of the limited number of losses, but because subjects (and investigators) were not informed of the level of DNA damage, which was assessed only during statistical analysis of data, minimizing the possibility that results are affected by selection bias.

Results on specific causes of death were meaningful only for most common causes such as cardiovascular diseases and malignant neoplasm. The strongest association was found for gastro-intestinal cancer, showing a suggestive parallelism with results from similar analyses reported for the micronucleus assay and chromosome aberrations^10–13^.

This study has a number of strengths, and several weaknesses. The major strength is the cohort design, which is the least affected by bias, especially the reverse causality bias, and has been used for the validation of several biomarkers before. It should be noted also that despite the evidence coming from cross-sectional studies, and the well-known mechanistic models, no epidemiological evidence has been published so far, demonstrating that the extent of DNA damage in healthy subjects may predict the risk of death and NCD, including cancer. As regards limitations, the small size of the cohort and the heterogeneity of the comet assay descriptors are surely the most critical, although the standardization based on tertiles is a well-established approach to deal with laboratory variability. Besides epidemiological weaknesses, there is the need to substantiate the mechanistic link between early genomic damage and such a long-term event as overall mortality. On the other hand, the presence of extensive evidence linking DNA damage to NCDs such as cardiovascular diseases and cancer, which in turn are the major causes of death, suggests a plausible pathway linking the extent of DNA damage to the risk of death. The inactivation of tumor suppressor genes or the amplification of oncogenes are well known consequences of DNA breakage, a process leading to the formation of the atherosclerotic plaque^34–36^.

Another limitation is the possible effect of a decline in DNA repair capacity associated with ageing, oxidative stress and inflammation, which was not evaluated. It should be noted that the DNA repair activity may also be influenced by the same exposures that cause DNA damage, i.e., smoking, dietary factors as well as genotoxic agents in the environment and occupational settings. Thus, upregulation of the DNA repair activity may occur concurrently with a high rate of genotoxic insults due to external exposures.

The results of this analysis suggest a new role for the comet assay as a tool to measure risk biomarkers. The sensitivity analysis that showed that DNA damage can predict the risk of death independently from the duration of the follow-up (Supplementary Table 2) encourages further research on the use of the comet assay in long-term strategies aimed at preventing the risk of diseases as well as in pre-clinical applications.

In conclusion, the findings of this study strengthen the existing evidence that the level of DNA damage in circulating leukocytes of healthy individuals may be predictive of the risk of chronic diseases and mortality, reflecting events such as accelerated aging, telomere capping loss, oxidative stress and more generally genomic instability. These results disclose a number of issues relevant to both ethics and public health policies. However, applicability at the individual level has still to be established because of the low absolute level of risk observed and the large inter-individual variability of these results, not forgetting the role of individual parameters such as DNA repair competence. Nevertheless, from now on the use of assays measuring DNA damage to predict the risk of NCD diseases and mortality in population groups exposed to environmental or occupational risks or bearing a susceptible genetic profile should be considered for inclusion in public health strategies.

## Material and methods

### Study design and cohorts

The study design replicated the approach followed by similar studies^10–16^. All 44 laboratories contributing data to the hCOMET database within the framework of the hCOMET EU COST Action CA15132 were invited to participate to a pooled historical cohort study. The criteria for inclusion of laboratories in the full-scale study included 1) having had data published in peer-reviewed literature reporting the comet assay protocol used, and 2) providing evidence that personal identification was available for all subjects and the mortality follow-up was feasible through linkage with national/local cause of death registries. Data on cancer incidence were recorded when contact with cancer registry was available. A detailed description of the protocol used for the assay was collected from each laboratory submitting a database and evaluated by the hCOMET steering committee for compliance to standard methodology^6^. In brief, subjects who underwent DNA damage screening with the comet assay applied to circulating leukocytes for biomonitoring purposes were followed from the date of the assay until the date of death, emigration, or the end of a defined follow-up period (December 2017–January 2019, depending on the country), whichever occurred first. The median duration of the follow-up was 10.8 years. Only adult subjects with valid demographic data, without a previous cancer diagnosis at the time of test were included. Overall, 2,403 subjects, corresponding to 25,978 person-years, were contributed to the hCOMET cohort by 16 laboratories from Brazil, Italy, Poland, Russia, Slovakia, Norway, Spain, Croatia, Serbia, Cuba, France, Turkey, and Hungary. All laboratories scored a minimum of 100 comets per individual sample, and all subjects were sampled only once. More detail on the characteristics of the cohort is reported in Table 1. In the large majority of the cohort (nearly 98%), information on cause-specific mortality was obtained through linkage with local/national registries or with hospital records. In the remaining 2% an active system of follow-up for mortality was set up via personal knowledge. Overall, 308 deaths were registered and coded using the ICD-IX classification system. Data on cancer incidence (151 cases) were extremely heterogeneous within different laboratories, with results substantially based on two laboratories, EU31 and EU33, and were affected by a generally poor quality of cancer diagnosis (40.1% of cases were assessed via medical records or by personal knowledge). The limited reliability of case-assessment, and the heterogeneous report of confounders by different centers prevented any further analyses of data relating to cancer incidence, which have not been reported among the results. All individual studies contributing data to the hCOMET cohort had already received approval from local ethical committees, and the informed consent for the collection and analysis of individual coded data was signed by all study subjects. The analysis of individual coded data was run in accordance with the Declaration of Helsinki and with the measures of the General Data Protection Regulation (EU) 2016/679 (GDPR). The pooled analysis of data was approved by the ethics committee of the IRCCS San Raffaele Roma, Rome, Italy (12 December 2015, Prot. N. 10/15), the centre coordinating data collection and running the statistical analysis of data.

## The hCOMET database

More detail about the study design and the quality procedures implemented in each study can be found in the original papers (Supplementary list of references) and summarized in the previous publications of the consortium,^5, 19, 37^ which included data from 19,350 subjects, contributed by 44 laboratories. In addition, available information was collected concerning demographic parameters, lifestyle, occupational exposure, smoking, diet, genetic profile, and diagnoses of chronic diseases. To take into consideration the extensive heterogeneity of methods used in different laboratories, special care has been given to the hCOMET questionnaire collecting protocol description and technical features. Separate analyses were performed for each descriptor of the comet assay, namely tail intensity (%T), tail length (TL), tail moment (TM), and visual scoring (VS) to compare the different sensitivity of the four descriptors. To standardize for the large interlaboratory variation, mostly due to exposure to genotoxic agents, cell type (isolated lymphocytes or whole blood), and sample processing (fresh/frozen)^19^ , all subjects were classified within each laboratory according to tertile frequency of each descriptor as follows: low (1–33 percentile), medium (34–66 percentile) or high (67–100 percentile). The approach based on tertiles was the most efficient according to the Akaike information criteria when compared in the pooled database with other methods of standardization such as z scores and deviation from the mean^13, 38^. To increase the number of events suitable for analysis, the %T sub-cohort, the descriptor with more observations, and generally considered the most reliable,^5, 37^, was enriched with data from other descriptors, creating a composite endpoint that represented the primary endpoint of the analysis. Whenever %T was not measured in the original studies, but other descriptors were available, the tertile of DNA damage was estimated using the mean value of tertiles from the other markers, rounded to the nearest integer. This approach was based on the assumption that all descriptors of the comet assay provide suitable measure DNA damage within each of the included studies.

## Individual studies.

The individual studies contributing data to the cohort are listed in the Supplementary list of references. Sample size ranged from 17 to 738 subjects, and studies were performed between 1996 and 2016. Information on age and sex was available for all subjects, while smoking status at the time of testing was available for 93.0% of the study subjects, with 32.2% declaring to have smoked during their life. The number of cigarettes smoked was available only for a small proportion of subjects. The number of subjects considered exposed to genotoxic agents or affected by disease in the original studies represented 66.8% of the whole cohort. Nine laboratories investigated various occupational exposures, four studied patients affected by chronic diseases, and three studied non exposed subjects. A description of all exposure groups by laboratory and by type of exposure/disease appears in the Supplementary Table 3.

## Statistical analysis

The Kaplan–Meier product-limit method was applied to estimate the survival time after comet assay testing by different tertiles of DNA damage. Survival curves were compared by the log-rank test^39^. The association between events and the tertile of DNA damage was modeled according to the Cox proportional hazard regression model, adjusting for age at test (years), sex (M/F), smoking habit (yes/no), and occupational exposure/disease (unexposed/controls; occupationally exposed, affected by disease). Diagnostic tests did not detect any substantial violation of underlying assumptions of the Cox regression. To evaluate whether the overall association between DNA damage and overall mortality was driven by a specific cohort, a sensitivity analysis was performed sequentially removing cohorts from the analysis. The potential effect modification due to the presence of an occupational exposure to DNA damaging agents or of a chronic disease was explored by means of the likelihood ratio test^40^. Due to the limited number of cases in the lowest and medium tertiles of unexposed/control subjects, the pool of all control subjects was used as the referent value for the interaction analysis. Additional analyses were performed by laboratory and by specific cause of death. Finally, to provide an additional control for confounding, data were analysed after applying the *propensity score matching*, a statistical matching technique that attempts to estimate the effect of exposure by accounting for the covariates that predict exposure. Results of this analysis overlapped almost perfectly those from traditional regression analysis (data not shown), and therefore only the latter have been reported. To test the possibility that the likely presence of chronic diseases could have influenced the risk associated with the tertile of DNA damage, we stratified the entire cohort according to the median follow-up time, i.e. 0–5 and > 5 years, and tested a possible effect modification due to time since test. Statistical packages SPSS for Windows (*IBM SPSS Statistics for Windows, version 26. IBM Corp., Armonk, N.Y., USA*), and STATA statistical software *(StataCorp. 2013. Stata Statistical Software: Release 13. College Station, TX: StataCorp LP*) were used for all analyses.

### Data and code availability

The database supporting the current study has not been deposited in a public repository because of restriction of the ethical clearance (sensitive information), but are available from the corresponding author on request.

## Supplementary Information

Supplementary Information.
