# Author Correction: DNA damage in circulating leukocytes measured with the comet assay may predict the risk of death

**DOI:** 10.1038/s41598-021-98620-6

**Published:** 2021-09-20

**Authors:** Stefano Bonassi, Marcello Ceppi, Peter Møller, Amaya Azqueta, Mirta Milić, Monica Neri, Gunnar Brunborg, Roger Godschalk, Gudrun Koppen, Sabine A. S. Langie, João Paulo Teixeira, Marco Bruzzone, Juliana Da Silva, Danieli Benedetti, Delia Cavallo, Cinzia Lucia Ursini, Lisa Giovannelli, Silvia Moretti, Patrizia Riso, Cristian Del Bo’, Patrizia Russo, Malgorzata Dobrzyńska, Irina A. Goroshinskaya, Ekaterina I. Surikova, Marta Staruchova, Magdalena Barančokova, Katarina Volkovova, Alena Kažimirova, Bozena Smolkova, Blanca Laffon, Vanessa Valdiglesias, Susana Pastor, Ricard Marcos, Alba Hernández, Goran Gajski, Biljana Spremo-Potparević, Lada Živković, Elisa Boutet-Robinet, Hervé Perdry, Pierre Lebailly, Carlos L. Perez, Nursen Basaran, Zsuzsanna Nemeth, Anna Safar, Maria Dusinska, Andrew Collins, for the hCOMET project, Diana Anderson, Vanessa Andrade, Cristiana Costa Pereira, Solange Costa, Kristine B. Gutzkow, Carina Ladeira, Massimo Moretti, Carla Costa, Irene Orlow, Emilio Rojas, Bertrand Pourrut, Marcin Kruszewski, Siegfried Knasmueller, Sergey Shaposhnikov, Bojana Žegura, Helga Stopper

**Affiliations:** 1 Unit of Clinical and Molecular Epidemiology, IRCCS San Raffaele Roma, Rome, Italy; 2 Department of Human Sciences and Quality of Life Promotion, San Raffaele University, Unit of Clinical and Molecular Epidemiology, IRCCS San Raffaele Roma, Via di Val Cannuta, 247, 00166 Rome, Italy; 3 Clinical Epidemiology Unit, San Martino Policlinic Hospital, Genoa, Italy; 4 Department of Public Health, Section of Environmental Health, University of Copenhagen, Oster Farimagsgade 5A, 1014 Copenhagen, Denmark; 5 Department of Pharmacology and Toxicology, University of Navarra, C/Irunlarrea 1, 31008 Pamplona, Spain; 6 C/Irunlarrea 3, IdiSNA, Navarra Institute for Health Research, 31008 Pamplona, Spain; 7 Mutagenesis Unit, Institute for Medical Research and Occupational Health, Ksaverska cesta 2, 10000 Zagreb, Croatia; 8 Department of Environmental Health, Section of Molecular Toxicology, Norwegian Institute of Public Health (NIPH), Lovisenberggt 6, 0456 Oslo, Norway; 9 Department of Pharmacology and Toxicology, School of Nutrition and Translational Research in Metabolism, University of Maastricht, Universiteitssingel 50, 6200 MD Maastricht, The Netherlands; 10 Flemish Institute of Technological Research, Environmental Risk and Health Unit VITO - BIOMo, Mol, Belgium; 11 Environmental Health Department, National Institute of Health, Rua Alexandre Herculano, 321, 4000-055 Porto, Portugal; 12 Environmental Health Department, Instituto Nacional de Saúde Doutor Ricardo Jorge, Rua Alexandre Herculano 321, 4000-055 Porto, Portugal; 13 EPIUnit - Instituto de Saúde Pública, Universidade Do Porto, Rua das Taipas, no 135, 4050-600 Porto, Portugal; 14 Laboratory of Genetic Toxicology, Lutheran University of Brazil (ULBRA), and La Salle University (UNILASALLE), Canoas, RS Brazil; 15 Department of Occupational and Environmental Medicine, Epidemiology and Hygiene (DiMEILA), Italian Workers’ Compensation Authority (INAIL), Via Fontana Candida 1, 00078 Monte Porzio Catone (Rome), Italy; 16 Department NEUROFARBA, University of Florence, Viale G. Pieraccini 6, 50139 Florence, Italy; 17 Department of Health Sciences, Division of Dermatology, University of Florence, Palagi Hospital, Viale Michelangelo 41, Florence, Italy; 18 Department of Food, Environmental and Nutritional Sciences (DeFENS), University of Milan, Via Celoria 2, 20133 Milan, Italy; 19 Department of Radiation Hygiene and Radiobiology, National Institute of Public Health NIH – National Research Institute, 24 Chocimska Street, 00-791 Warsaw, Poland; 20 Laboratory for the Study of the Pathogenesis of Malignant Tumors, National Medical Research Center for Oncology, 14 line 63, 344037 Rostov-on-Don, Russia; 21 Institute of Biology, Medical Faculty, Slovak Medical University, Limbova 12, 83303 Bratislava, Slovakia; 22 Cancer Research Institute, Biomedical Research Center of the Slovak Academy of Sciences, Dubravska cesta 9, Bratislava, Slovakia; 23 Grupo DICOMOSA, Centro de Investigaciones Científicas Avanzadas (CICA), Departamento de Psicología, Facultad de Ciencias de La Educación, Universidade da Coruña, Campus Elviña s/n, 15071 A Coruña, Spain; 24 Instituto de Investigación Biomédica de A Coruña (INIBIC), AE CICA-INIBIC, Oza, 15071 A Coruña, Spain; 25 Grupo DICOMOSA, Centro de Investigaciones Científicas Avanzadas (CICA), Departamento de Biología, Facultad de Ciencias, Universidade da Coruña, Campus A Zapateira s/n, 15071 A Coruña, Spain; 26 Department of Genetics and Microbiology, Faculty of Biosciences, Universitat Autònoma de Barcelona, 08193 Cerdanyola del Vallès (Barcelona), Spain; 27 Consortium for Biomedical Research in Epidemiology and Public Health (CIBERESP), Carlos III Institute of Health, 28029 Madrid, Spain; 28 Center of Biological Research, Faculty of Pharmacy, University of Belgrade, VojvodeStepe 450, Belgrade, Serbia; 29 Toxalim (Research Centre in Food Toxicology), Université de Toulouse, INRAE, ENVT, INP-Purpan, UPS, Toulouse, France; 30 Univ Paris-Saclay, CESP, Villejuif, France; 31 ANTICIPE Unit, INSERM & University of Caen-Normandie Centre François Baclesse, Avenue du Général Harris, 14076 Caen Cedex 05, France; 32 Department of Biochemistry, Instituto de Ciencias Básicas Y Preclínicas “Victoria de Giron”, Universidad de Ciencias Médicas de La Habana, 146 St. and 31 Ave, No, 3102 Playa, Habana, Cuba; 33 Department of Pharmaceutical Toxicology, Faculty of Pharmacy, Hacettepe University, Ankara, Turkey; 34 Department of Non-Ionizing Radiation, National Public Health Center, Anna Street 5, 1221 Budapest, Hungary; 35 NILU, Health Effects Laboratory, Kjeller, Norway; 36 Department of Nutrition, Institute of Basic Medical Sciences, University of Oslo, Sognsvannsveien 9, 0372 Oslo, Norway; 37 University of Bradford, Bradford, UK; 38 University of Southern Santa Catarina, Santa Catarina, Brazil; 39 National Institute of Health Doutor Ricardo Jorge, Porto, Portugal; 40 Norwegian Institute of Public Health, Olso, Norway; 41 Instituto Politécnico de Lisboa, Universidade NOVA, Lisbon, Portugal; 42 University of Perugia, Perugia, Italy; 43 Memorial Sloan Kettering Cancer Center, New York, USA; 44 Universidad Nacional Autónoma de México, Mexico City, Mexico; 45 ISA Lille, Université de Toulouse, Toulouse, France; 46 Institute of Nuclear Chemistry and Technology, Warszawa, Poland; 47 Medical University of Vienna, Wien, Austria; 48 University of Oslo, Olso, Norway; 49 National Institute of Biology, Ljubljana, Slovenia; 50 University of Würzburg, Würzburg, Germany

Correction to: *Scientific Reports* 10.1038/s41598-021-95976-7, published online 18 August 2021

The original version of this Article contained errors.

The spelling of the author Monica Neri was incorrectly given as Neri Monica.

Additionally, Affiliation 39 was incorrectly given as ‘National Institute of Health, Lisbon, Portugal’. The correct affiliation is listed below:

National Institute of Health Doutor Ricardo Jorge, Porto, Portugal.

The original Article has been corrected.

