# Supplementary material for: DNA damage in circulating leukocytes measured with the comet assay may predict the risk of death
Source: Sci Rep. 2021 Aug 18;11:16793. doi: 10.1038/s41598-021-95976-7 (PMC8373872; doi:10.1038/s41598-021-95976-7)
Supplement: Supplementary file 1 — Supplementary Information. [file 41598_2021_95976_MOESM1_ESM.docx]

**Supplementary references.** List of published studies contributing data to the cohort study

Benedetti D, Nunes E, Sarmento M, Porto C, Dos Santos CE, Dias JF, da Silva J. Genetic damage in soybean workers exposed to pesticides: evaluation with the comet and buccal micronucleus cytome assays. Mutat Res. 2013 752:28-33.

Benedetti D, Lopes Alderete B, de Souza CT, Ferraz Dias J, Niekraszewicz L, Cappetta M, Martínez-López W, Da Silva J. DNA damage and epigenetic alteration in soybean farmers exposed to complex mixture of pesticides. Mutagenesis. 2018 33:87-95.

Garaj-Vrhovac V, Gajski G, Brumen V. Alkaline comet assay as a biomarker of DNA-damage encountered in workers engaged in cigarette manufacturing. Period. Biol. 2009; 11(1): 85-90.

Garaj-Vrhovac V, Gajski G, Pažanin S, Sarolić A, Domijan AM, Flajs D, Peraica M. Assessment of cytogenetic damage and oxidative stress in personnel occupationally exposed to the pulsed microwave radiation of marine radar equipment. Int J Hyg Environ Health. 2011; 214(1): 59-65.

Lebailly P Mirey G Herin F Lecluse Y Salles B Boutet Robinet E . DNA damage in B and T lymphocytes of farmers during one pesticide spraying season. Int Arch Occup Environ Health (2015) 88:963–972

Ursini CL, Cavallo D, Colombi A, Giglio M, Marinaccio A, Iavicoli S. Evaluation of early DNA damage in healthcare workers handling antineoplastic drugs. Int Arch Occup Environ Health. 2006 Nov;80(2):134-40

Cavallo D, Fresegna AM, Ciervo A, Maiello R, Buresti G, Iavicoli S. Genotoxic effects in hospital Laboratory workers exposed to complex chemical mixtures. Toxicology Letters vol.189, Supplement 1, pag. S153, 2009

Pitozzi V, Giovannelli L, Bardini G, Rotella CM, Dolara P. Oxidative DNA damage in peripheral blood cells in type 2 diabetes mellitus: higher vulnerability of polymorphonuclear leukocytes. Mutat Res. 2003 Aug 28;529(1-2):129-33.

Pacini S, Giovannelli L, Gulisano M, Peruzzi B, Polli G, Boddi V, Ruggiero M, Bozzo C, Stomeo F, Fenu G, Pezzatini S, Pitozzi V, Dolara P. Association between atmospheric ozone levels and damage to human nasal mucosa in Florence, Italy. Environ Mol Mutagen. 2003;42(3):127-35.

Giovannelli L, Pitozzi V, Moretti S, Boddi V, Dolara P. Seasonal variations of DNA damage in human lymphocytes: correlation with different environmental variables. Mutat Res. 2006 Jan 29;593(1-2):143-52. Epub 2005 Aug 10.

Riso P, Klimis-Zacas D, Del Bo' C, Martini D, Campolo J, Vendrame S, Møller P, Loft S, De Maria R, Porrini M. Effect of a wild blueberry (Vaccinium angustifolium) drink intervention on markers of oxidative stress, inflammation and endothelial function in humans with cardiovascular risk factors.Eur J Nutr 2013;52:949–961.

Riso P, Martini D, Møller P, Loft S, Bonacina G, Moro M, Porrini M. DNA damage and repair activity after broccoli intake in young healthy smokers. Mutagenesis 2010; 25:595–602.

Del Bo' C, Porrini M, Campolo J, Parolini M, Lanti C, Klimis-Zacas D, Riso P. A single blueberry (Vaccinium corymbosum) portion does not affect markers of antioxidant defence and oxidative stress in healthy volunteers following cigarette smoking. Mutagenesis 2016, 31:215-24.

Russo P, Lamonaca P, Milic M, Rojas E, Prinzi G, Cardaci V, Vitiello L, Proietti S, Santoro A, Tomino C, Fini M, Bonassi S. Biomarkers of DNA damage in COPD patients undergoing pulmonary rehabilitation: Integrating clinical parameters with genomic profiling.

Mutat Res. 2019, 843:111-117.

Dobrzynska MM, Pachocki KA, Gajowik A, Radzikowska J, Sackiewicz A: The effect of occupational exposure to ionizing radiation on the DNA damage in peripheral blood leukocytes of nuclear medicine personnel. J Occupat Health 2014, 56, 379-386.

Sirota NP, Kuznetsova EA. Spontaneous DNA damage in peripheral blood leukocytes from donors of different age. Bull Exp Biol Med. 2008, 145:194-7.

Surikova E.I., Goroshinskaya I.A., Frantsiyants E.M., Tarnopolskaya O.V., Sirota N.P., Vladimirova L.Yu., Tikhanovskaya N.M., Kuznetsova E.А. Comet assay in evaluation of individual DNA-damage of breast cancer patients undergoing chemotherapy with doxorubicin-contaio9ni9ng regimens.12th International Comet Assay Workshop (Pamplona, Spain, August 29-31, 2017). Book of Abstracts 12th ICAW 2017, Oral Presentation O36, P.56 - Nutritional and clinical aspects.

Dusinská M1, Barancoková M, Kazimírová A, Harrington V, Volkovová K, Staruchová M, Horská A, Wsólová L, Collins A. Does occupational exposure to mineral fibres cause DNA or chromosome damage? Mutat Res. 2004, 553:103-10.

Dusinska M1, Staruchova M, Horska A, Smolkova B, Collins A, Bonassi S, Volkovova K. Are glutathione S transferases involved in DNA damage signalling? Interactions with DNA damage and repair revealed from molecular epidemiology studies. Mutat Res. 2012, 736:130-7.

Laffon, B., Aguilera, F., Ríos-Vázquez, J., Valdiglesias, V., Pásaro, E. Follow-up study of genotoxic effects in individuals exposed to oil from the tanker Prestige, seven years after the accident. Mutation Research 23014, 760: 10-16.

Laffon, B., Pásaro, E., Méndez, J. Evaluation of genotoxic effects in a group of workers exposed to low levels of styrene. Toxicology 2002, 171: 175-186.

Laffon, B.; Teixeira, J.P.; Silva, S.; Loureiro, J.; Torres, J.; Pásaro, E.; Méndez, J.; Mayan, O. Genotoxic effects in a population of nurses handling antineoplastic drugs, and relationship with genetic polymorphisms in DNA repair enzymes. American Journal of Industrial Medicine 2005, 48: 128-136.

Laffon, B., Teixeira, J.P., Silva, S., Roma-Torres, J., Perez-Cadahia, B., Mendez, J., Pasaro, E., Mayan, O. Assessment of occupational genotoxic risk in the production of rubber tyres. Ann Occup Hyg 2006, 50: 583-592.

Corredor Z, Rodríguez-Ribera L, Silva I, Díaz JM, Ballarín J, Marcos R, Coll E, Pastor S. Levels of DNA damage in peripheral blood lymphocytes of patients undergoing standard hemodialysis vs on-line hemodiafiltration: A comet assay investigation Mutat Res. 2016, 808:1-7.

Stoyanova E, Pastor S, Coll E, Azqueta A, Collins AR, Marcos R. Base excision repair capacity in chronic renal failure patients undergoing hemodialysis treatment. Cell Biochem Funct. 2014, 32:177-82.

Kogevinas M, Villanueva CM, Font-Ribera L, Liviac D, Bustamante M, Espinoza F, Nieuwenhuijsen MJ, Espinosa A, Fernandez P, DeMarini DM, Grimalt JO, Grummt T, Marcos R. Genotoxic effects in swimmers exposed to disinfection by-products in indoor swimming pools. Environ Health Perspect. 2010, 118:1531-7.

Čabarkapa A., Živković L., Borozan S., Zlatković-Švenda M., Dekanski D., Jančić I., Radak-Perović M., Bajić V., and Spremo-Potparević B. Dry Olive Leaf Extract in Combination with Methotrexate Reduces Cell Damage in Early Rheumatoid Arthritis Patients—A Pilot Study. Phytotherapy Research, 2016, 30: 1615-1623.

Ündeğer, Ü., Başaran, N., Kars, A., Güç, D.:Assessment of DNA Damage in Nurses Handling Antineoplastic Drugs by the Alkaline COMET Assay. Mutation Research, 1999, 439, 277-285.

**Supplementary Table 1.** Overall mortality risk by tertile of DNA damage measured with the comet assay (Composite endpoint). Interaction analysis by occupational exposure/disease using medium tertile of controls as reference level.

|  | **Deaths**  **(no.)** | **Subjects**  **(no.)** | **HR** | **95% CI** | ***P*-value** |
| --- | --- | --- | --- | --- | --- |
| **Controls** |  |  |  |  |  |
| *Low tertile* | 1 | 264 | 0.67 | 0.06-7.36 | 0.741 |
| *Medium tertile* | 2 | 269 | *ref.* | - | - |
| *High tertile* | 10 | 248 | 6.07 | 1.33-27.7 | 0.020 |
| **Occupationally**  **exposed** |  |  |  |  |  |
| *Low tertile* | 12 | 349 | 2.78 | 0.60-12.8 | 0.190 |
| *Medium tertile* | 12 | 314 | 2.98 | 0.65-13.7 | 0.161 |
| *High tertile* | 17 | 326 | 3.86 | 0.87-17.2 | 0.077 |
| **NCD Patients** |  |  |  |  |  |
| *Low tertile* | 65 | 208 | 28.1 | 6.67-118.1 | <0.001 |
| *Medium tertile* | 82 | 204 | 35.5 | 8.52-148.0 | <0.001 |
| *High tertile* | 107 | 204 | 36.5 | 8.81-151.5 | <0.001 |

*Estimates adjusted for age, sex, smoking habit. For some predictors the sum of the frequencies of each categories does not amount to the overall due to missing values.*

**Supplementary Table 2.** Overall mortality risk by tertile of DNA damage measured with the comet assay (composite endpoint) by years of follow-up.

| **DNA damage** | Follow-up | |
| --- | --- | --- |
|  | 0-5 years | >5 years |
|  | **Hazard ratio**  **(95% CI)** | **Hazard ratio**  **(95% CI)** |
| *Low tertile*  *Medium tertile*  *High tertile* | 1.00  1.11  (0.74-1.65)  1.36  (0.93-2.00) | 1.00  1.45  (0.89-2.34)  1.53  (0.96-2.44) |

**: adjusted by age, sex, smoking habit and exposure*

**Supplementary Table 3.** Description of Exposure/disease studied in single studies contributing data to the hCOMET cohort study

| **Country** | **Laboratory** | **Exposure code^*^** | **Exposure/Disease** |
| --- | --- | --- | --- |
| Brazil | CSA7 | E | Pesticides |
| Italy | EU15 | E | Antineoplastic drugs |
| Italy | EU17 | D | Diabetes II; post-menopausal women |
| Italy | EU18 | - | Dietary supplementation |
| Italy | EU19 | D | COPD |
| Poland | EU23 | E | Ionizing radiation |
| Russia | EU27 | - | Healthy controls |
| Slovakia/Norway | EU31 | E | Fibers |
| Spain | EU32 | E | Oil; styrene; antineoplastic drugs |
| Spain | EU33 | D | Chronic kidney disease |
| Croatia | EU4 | E | Cigarette manufacturing; non-ionizing radiation; healthy controls |
| Serbia | EU42 | D | Rheumatoid arthritis |
| France | EU8 | E | Pesticides |
| Cuba | CSA2 | E | Ionizing radiation |
| Turkey | EU34 | E | Antineoplastic drugs |
| Hungary | EU50 | - | Healthy controls |

***^*^*** *= E (Exposure), D (Disease)*
